# Comparison of Subacromial Bursitis on the Shoulder of the Same Individual After Superior Capsule Reconstruction Using Allograft and Rotator Cuff Tear: A Case Report

**DOI:** 10.7759/cureus.58260

**Published:** 2024-04-14

**Authors:** Mitsuyoshi Matsumoto, Tomonori Kenmoku, Ryo Tazawa, Kosuke Inoue, Masashi Takaso

**Affiliations:** 1 Orthopaedic Surgery, Kitasato University Hospital, Sagamihara, JPN; 2 Orthopaedic Surgery, Kitasato University Hospital, Sagamihara City, JPN

**Keywords:** safety, pathology, allograft, superior capsular reconstruction, rotator cuff tears

## Abstract

This paper reports a pathological comparison between the synovium of the shoulder with rotator cuff tears (RCTs) with or without an allograft in the same patient and assesses allograft remodeling after superior capsular reconstruction (SCR). A 49-year-old man underwent SCR with a fascia lata allograft for irreparable RCTs. Two years postoperatively, the patient underwent arthroscopic rotator cuff repair for left RCTs and arthroscopic debridement to alleviate right shoulder pain. Additionally, revascularization was confirmed in the allograft of the fascia lata. In conclusion, allografts are considered highly safe and expected to be engrafted after SCR.

## Introduction

Superior Capsule Reconstruction (SCR) is effective in patients with rotator cuff tears (RCTs) that are not repairable in a primary setting [1]. SCR requires the use of an autograft. However, recent reports have shown that SCR using allografts has good clinical outcomes [2,3]. Allografts can be an effective alternative to autografts [4]. However, the safety and remodeling of allografts after SCR remain unclear. This report presents the pathological findings of the synovial tissue obtained from a shoulder with an allograft and a shoulder without an allograft in the same patient on the same day and reports the features of allograft remodeling observed two years after SCR.

## Case presentation

**Figure 1:**
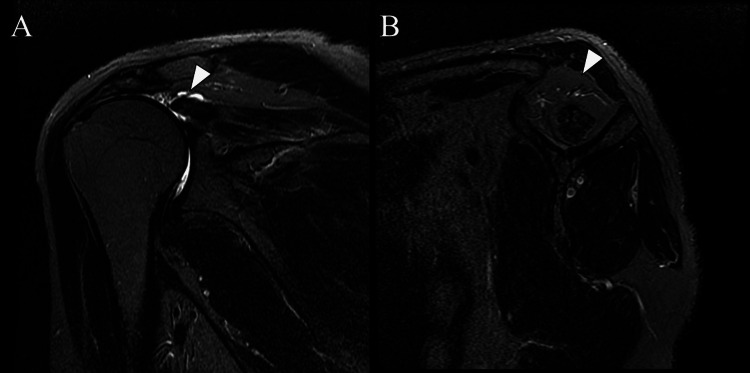
Preoperative magnetic resonance imaging of the right shoulder (A) Fat-suppressed T2-weighted image obtained in the oblique coronal plane shows massive rotator cuff tears at the level of the supraspinatus tendon; (B) Fat-suppressed T2-weighted image obtained in the sagittal plane showing advanced fatty infiltration of the supraspinatus muscle.

A 49-year-old man experienced severe right shoulder pain, night pain, and limited range of motion (ROM) for approximately one year before referral. The pain gradually increased, and recalcitrant pain at night also increased. He was also receiving treatment for uncontrollable epilepsy (seizures: 2-3 episodes monthly). On the first visit, his Constant-Murley score was 13 points [5]. Magnetic resonance imaging (MRI) revealed massive RCTs in the right shoulder with advanced fatty infiltration of the supraspinatus muscle (Figure 1) [6].

Considering the size of the tear, advanced fatty infiltration, age, and medical history, we performed arthroscopic SCR with an allograft of the fascia lata. The surgery was essentially based on the technique reported by Mihata et al. [7]. We could not find the acetabularization at the acromion before surgery, although the superior migration was confirmed. The bone spur of the acromion was not exited. Therefore, we only perform the synovectomy under the acromion. This allograft was harvested according to the guidelines of the Japanese Society of Tissue Transplantation after the death of a patient who had consented before life. An allograft of the fascia lata was harvested and stored at −80°C for >3 months. After three months, the allograft was removed from the surrounding tissues. After washing with copious amounts of water, the graft was soaked in 0.3 mg/ml of kanamycin for one hour at room temperature (20°C). Subsequently, this graft was dried at room temperature overnight and stored at −80°C.

**Figure 2:**
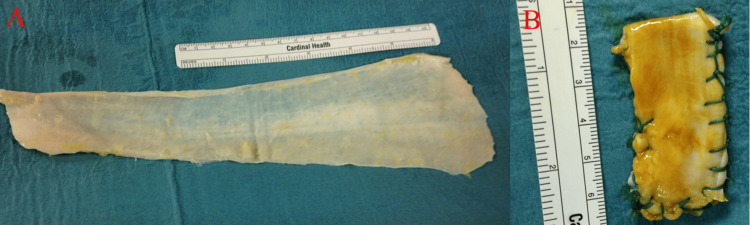
Allograft of fascia lata (A) Fascia lata allograft before trimming; (B) Trimmed allograft.

After thawing for 20 minutes at room temperature (20°C), the allograft of the fascia lata was trimmed intraoperatively. To prevent displacement, the transplanted tendon allograft was triple woven and stitched using No. 2 Ethibond sutures (Ethicon, Somerville, NJ). Thus, it was over 5.5 mm thick and was 5.5 × 2.0 cm in size (Figures 2A, 2B). The glenoid side was fixed with three knotless suture anchors (Juggerknot 1.5 mm, Zimmer-Biomet, Indiana, USA) around the glenoid. The greater tubercle of the humerus side was fixed with a 2 × 2 bridging suture (medial anchor, TWINFIX Ti 3.5-mm suture anchor, Smith and Nephew, Watford, UK; lateral anchor, VERSALOK BR 4.75, DePuy Synthes, MA, USA). The right shoulder was fixed with a shoulder brace for six weeks postoperatively. Physical therapy commenced two days postoperatively and passive and active shoulder ROM exercises were started at 35 and 42 days after surgery, respectively.

**Figure 3:**
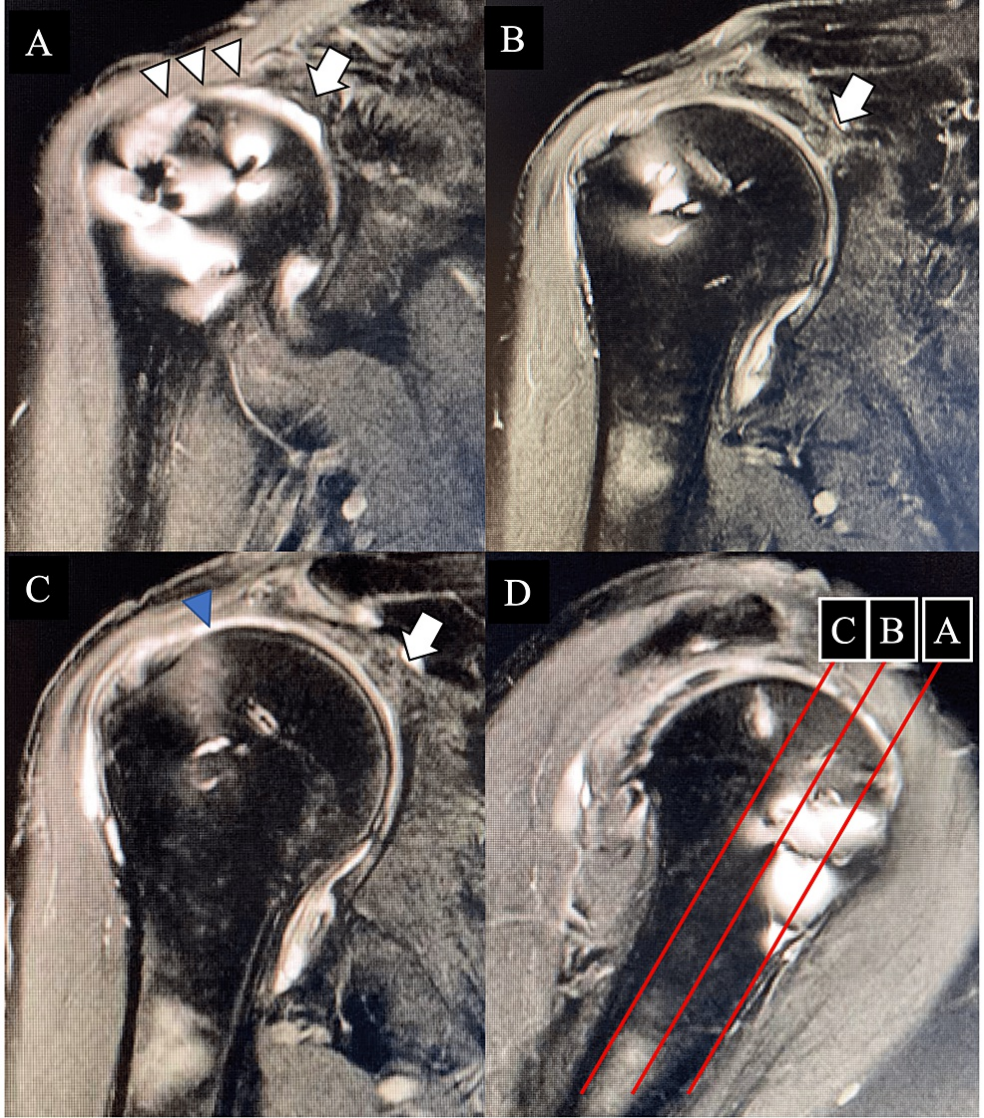
Magnetic resonance imaging of the right shoulder at 21 months after the primary surgery The oblique coronal (A-C) plane shows allograft engraftment was obtained at the glenoid side (white arrow). However, the allograft at the humeral side is considered to be partially ruptured (white triangle, considered engraftment area; blue triangle, ruptured area) (D) Red lines showed each coronal slice.

The postoperative outcomes were positive, although the patient continued to experience seizures 2-3 times/month. At 21 months postoperatively, the patient’s Constant-Murley score was 94 points; however, MRI revealed a re-ruptured grafted tendon at the humeral side (Figure 3).

At 25 months postoperatively, he visited our hospital due to pain in both shoulders. His right and left shoulders had limited ROM, with active flexion of 90° and 60° and active abduction of 75° and 60°, respectively. He experienced pain during movement and at night in both shoulders. The Constant-Murley of his right and left shoulders were 29 and 19 points, respectively.

**Figure 4:**
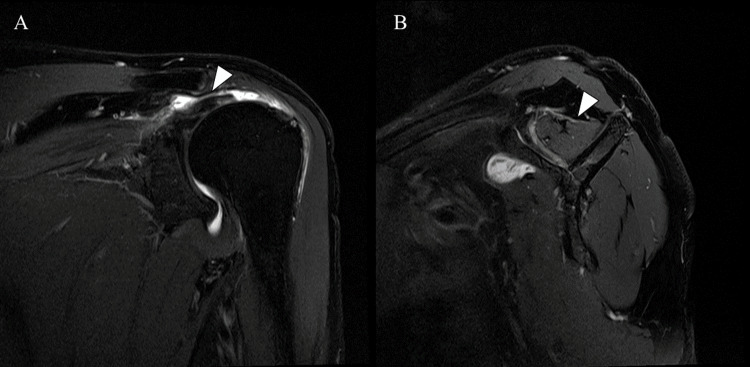
Preoperative magnetic resonance imaging of the left shoulder (A) Fat-suppressed T2-weighted images obtained in the oblique coronal plane shows moderate rotator cuff tears at the level of the supraspinatus tendon; (B) Fat-suppressed T2-weighted images obtained in the sagittal plane showing mild fatty infiltration of the supraspinatus muscle.

An MRI of the right shoulder at 25 months post-operation did not reveal advanced partial rupture of the allograft on the greater tubercle side compared to the MRI performed at 21 months postoperatively. However, the MRI of the left shoulder revealed medium-sized RCTs with minimal fatty infiltration (Figures 4A, 4B).

This patient had both shoulder pain and disabilities in activities of daily life at the same level. Therefore, we tried to perform at the same time. However, we need to immobilize both shoulders of this patient for four weeks after surgery, if we perform rotator cuff repair of reconstruction. We believed that his right shoulder could be managed using only arthroscopic debridement, considering the absence of advanced partial re-tears in the MRI findings between 21 and 26 months (Figure 3). We performed arthroscopic debridement of the right shoulder and arthroscopic rotator cuff repair (ARCR) for the left RCTs on the same day.

**Figure 5:**
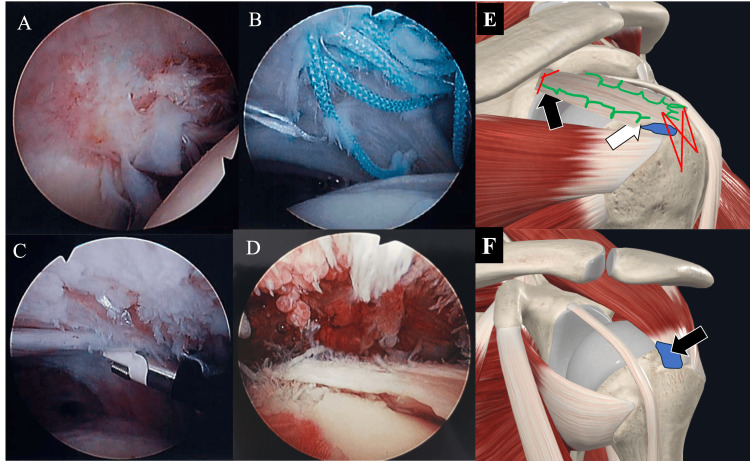
Intraoperative findings at the second surgery Grafted tendons, small blood vessels (black triangle) (A), and infiltration of inflammatory cells around Ethibond sutures (white triangle) (B) were observed in grafted tendons. The inflammatory infiltrate of the left shoulder (C) is more severe than the right shoulder (D). (E) Shema of right shoulder: Blue area, re-rapture area; Green line, Allograft stitch; Red line, Anchor thread; Black arrow, the position of Figure 5A; White arrow, the position of Figures 5B, 5C (F) Shema of left shoulder: Blue area, rotator cuff torn area; Black arrow, the position of Figure 5D.

We initiated surgery on the right shoulder and observed mild synovitis. The grafted tendon was firmly attached to the glenoid side. We could confirm vascularization in the grafted tendon (Figure 5A). Although the grafted tendon was partially torn at the middle facet of the greater tuberosity, the allograft was partially attached at the anterior part of the greater tuberosity (Figure 5B). Three samples were obtained from the site with the most notable features of synovitis at the subacromial bursa. We also debrided the detached area of the allograft around the greater tuberosity and retained it as a specimen. After obtaining the synovium and some of the unstable grafted tendons, we performed a synovectomy at the subacromial space and removed all exposed sutures at the footprint. We debrided the ruptured allograft around the middle facet and preserved the grafted tendon around the superior facet where engraftment was obtained (Figure 5C). Subsequently, ARCR on the left shoulder was performed. Subacromial synovitis was more severe in the left shoulder than in the right shoulder (Figure 5D). Three samples were obtained from the sites with the most notable features of synovitis at the subacromial bursa and rotator intervals. We performed ARCR using bridging sutures (medial anchor, HEALICOIL 5.5-mm knotless anchor, Smith and Nephew, Watford, UK; lateral anchor, VERSALOK BR 4.75, DePuy Synthes). The synovia of both shoulders and re-ruptured allografts were assessed as pathological specimens.

Postoperatively, active motion of the right shoulder was allowed after one week of immobilization with a sling. The left shoulder was immobilized with a brace for four weeks, and active ROM exercises were commenced four weeks postoperatively. The patient felt pain in his right shoulder, but the pain experienced at night, along with right shoulder function improved one week postoperatively. One year after the second surgery, his active ROMs of flexion in the right and left shoulders were 170° and 175°, respectively. Additionally, his active abduction angles were 170° and 175°, respectively. The right and left external rotation were improved to 70° and 75°, respectively. In the internal rotation, the spinal attainment levels of the thumb were at the L1 and Th10 levels, respectively. There was no pain during movement or at night in either shoulder. Constant-Murley scores of his right and left shoulders were 94 and 96 points, respectively.

The entire allograft specimen and synovial specimens were fixed in 20% neutral-buffered formalin. Some 4-μm-thick tissue sections were obtained and stained with haematoxylin and eosin. Histologic slides were examined using an OLYMPUS Microscope BX (Olympus, Tokyo, Japan) with an objective magnification of 10X, and images were captured using an OLYMPUS camera (DP72 and Software cell Sens, Standard version 1.5.).

**Figure 6:**
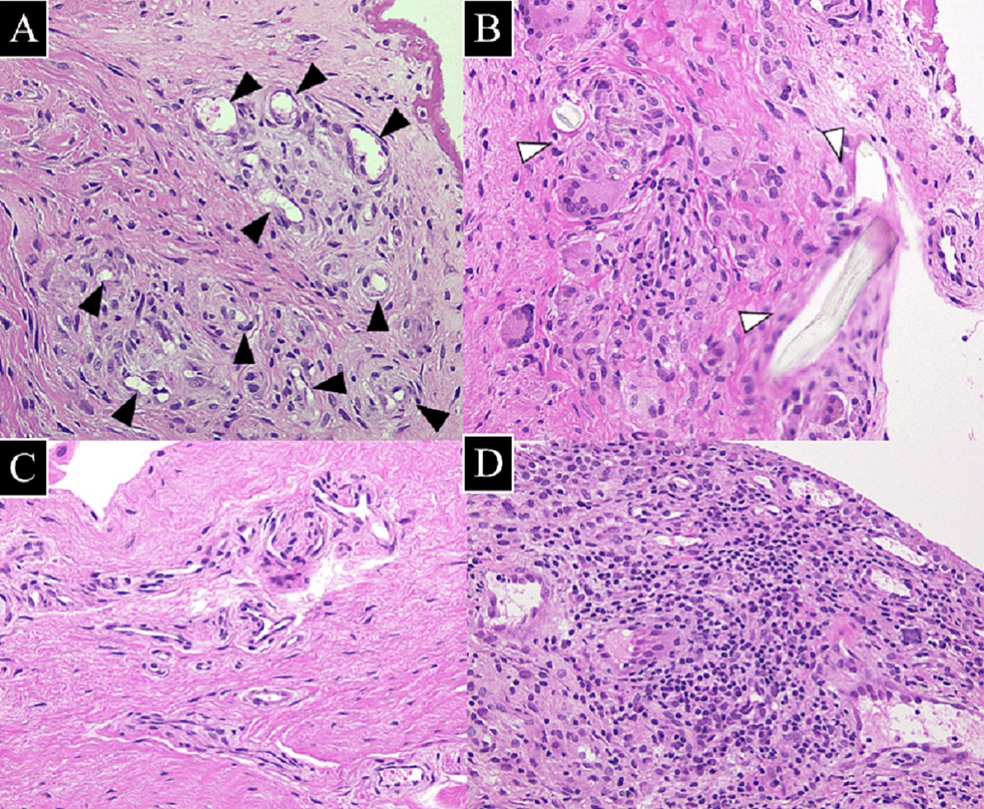
Histopathology images of left and right shoulder synovium (A-C) Pathology of the synovium of the right shoulder  (D) Pathology of the synovium of the left shoulder Small blood vessels (A, black arrow) and infiltration of inflammatory cells around Ethibond sutures.  Small blood vessels(B, white arrow) were observed in the grafted tendon. The inflammatory infiltrate of the left shoulder (D) is more severe than the right shoulder (C).

The pathological findings of the specimens obtained intraoperatively are shown in Figure 6. Moreover, 1.2 x 1.2 x 0.3 cm^3^ specimens were obtained. Small blood vessel growth was observed in the removed allograft. Additionally, aggregated macrophages and multinucleated and foreign giant cells with irregularly arranged nuclei and cytoplasmic acidophilic giant cells were identified (Figures 6A, 6B).

The inflammatory infiltrate appeared to be slightly increased; however, few perivascular-located lymphocytes and plasma cells were observed in the right subacromial bursa. Compared with the right shoulder, the subacromial synovium of the left shoulder showed more enhanced lymphocytic infiltration. Therefore, we defined the grades of the right and left shoulders as low and high grades of the inflammatory infiltrate of the synovitis, respectively (Figures 6C, 6D) [8]. The patient was informed that data concerning the case would be submitted for publication, and he provided written consent. The experimental protocol adhered to the institutional guidelines and received approval from the Clinical Research Review Board of the Kitasato Institute. Written informed consent was obtained from this patient.

## Discussion

Although allografts are recognized as valuable and safe materials for tendon and ligament reconstruction, tendon allografts have the potential risk of rejection due to immune incompatibility between the donor and recipient [2,4,9,10]. Immunological rejection after transplantation of allograft tendons is induced by tenocytes (not collagen tissues [11]). However, freeze-thawing can reduce the immunological antigenicity of tenocytes [4,8,12]. The processing procedure at our facility involves freezing the allograft at −80 °C before and after treatment with kanamycin sulphate during storage, which can help eliminate allogeneic graft tenocytes and prevent rejection after transplantation.

In our case, synovial membranes were obtained from both shoulders on the same day. Based on the assessment by Krenn et al., the grade of the synovitis in the subacromial bursa of the right shoulder with allograft was lower than that in the left shoulder without allograft [8]. This suggests that the excessive inflammation was not induced by subacromial impingement in the failed SCR using an allograft. Allograft did not provoke a marked inflammatory response contrary to the inflammation of the shoulder with RCTs. Therefore, the allograft is considerably safe and helpful for SCR, although only a single case was reported.

The allograft serves as a valuable matrix for reconstruction material. We believe the quality of a fascia lata allograft is better than that of a dermal graft because a fascia lata is the same musculotendinous tissue as the original technique [1,7]. However, no literature has been identified that provides histological assessments subsequent to the implementation of an allograft of the fascia lata. Dermal grafts are frequently employed in lieu of autografts for SCR, with previous studies attesting to their safety [13,14]. Nevertheless, a review article has outlined pertinent foundational research suggesting the occurrence of multinucleated giant cell and mononuclear cell reactions in an animal model post-dermal graft application [15]. Therefore, in instances where patients report enduring shoulder discomfort following SCR utilizing a noncellular allograft, clinicians should consider foreign body reaction in the differential diagnosis as a precautionary measure.

Remodelling and engraftment of autografts in SCR were previously undocumented but were observed in this instance. Utilizing MRI and arthroscopic observation, we successfully traced the trajectory of the allograft from the scapular side to the humerus side. Revascularization of the graft was also pathologically confirmed, indicating partial engraftment of the allograft into autologous tissue. In anterior cruciate ligament reconstruction, reports indicate that the remodelling and revascularization of allografts parallel the process observed in autografts [16]. This implies that histological changes in the graft may progress in a manner akin to those in autologous tissue. Within case reports of SCR using dermal graft, there is documentation supporting the engraftment of allografts with autologous tissue, even when the tissues differ [17]. This suggests that allografts may possess sufficient survival ability in the implanted area.

In our case, right shoulder pain and function improved after debridement of the unstable allograft and suture threads at the footprint. Therefore, we did not perform the acromial decompression. Of course, it is better for the patient if all the grafted tendons are viable [1,7]. However, considering the clinical outcome of the patient after SCR, some have reported no significant difference in pain and shoulder function between the healed and failed grafts [18,19]. Moreover, decreasing the tuberosity-to-acromion contact can lead to decreased pain and improved shoulder function [18,20]. The degree of inflammation was not severe; therefore, subacromial inflammation was difficult to be the cause of re-rupture. Consequently, mechanical stress is believed to be the primary factor contributing to the allograft's partial re-rupture, although the acetabularization of the acromion and a bone spur were not confirmed before and after SCR. Additionally, this right shoulder improvement after debridement was attributed to partial allograft engraftment and decreased stimulation by the unstable allograft and threads that existed between the acromion and tuberosity during elevation.

## Conclusions

The findings of our case support the potential safety and effectiveness of allografts in SCR and may represent a useful alternative to autografts. The study also underscores the importance of careful patient selection, processing techniques, and ongoing monitoring in optimizing outcomes with allografts in SCR. Furthermore, it is imperative to remain mindful of the potential risk of allograft rejection reaction. To confirm the safety and clinical outcome of SCR with grafts, the number of cases for pathological evaluation should be increased and long-term follow-up of each case should be conducted.
